# Web-Based Respondent-Driven Sampling to Assess Biobehavioral Factors Among Men Who Have Sex With Men in Thailand: Cross-Sectional Study

**DOI:** 10.2196/58076

**Published:** 2024-12-06

**Authors:** Watcharapol Srinor, Suvimon Tanpradech, Panupit Thiengtham, Samart Karuchit, Charif Naksuk, Thitipong Yingyong, Thananda Naiwatanakul, Sanny Northbrook, Wolfgang Hladik

**Affiliations:** 1 Division of Epidemiology, Department of Disease Control, Ministry of Public Health, DDC 10 Building, Tiwanon Road, Mueang, Nonthaburi, 11000, Thailand, 66 877711666; 2 Division of Global HIV & TB, U.S. Centers for Disease Control and Prevention, Nonthaburi, Thailand; 3 Informatics Section, Business Services Office, U.S. Centers for Disease Control and Prevention, Nonthaburi, Thailand; 4 Division of Global HIV & TB, U.S. Centers for Disease Control and Prevention, Atlanta, GA, United States

**Keywords:** online respondent-driven sampling, HIV, men who have sex with men, MSM, Bangkok, health clinic, public health, testing, stigma, online testing, HIV prevention, research data collection

## Abstract

**Background:**

Respondent-driven sampling (RDS) is the current standard for sampling key populations at risk for HIV infections but is usually limited to local implementation in single towns or cities. Web-based sampling eliminates this spatial constraint but often relies on self-selected convenience samples. We piloted a web-based RDS survey with biomarker collection among men who have sex with men (MSM) in Thailand.

**Objective:**

This study aimed to evaluate and demonstrate the feasibility of implementing a web-based RDS survey as a routine surveillance system in Thailand. The goal was to enhance surveillance efforts targeting hard-to-reach populations in the country.

**Methods:**

We developed a website to fully function like a conventional RDS survey office, including coupon verification, eligibility screening, consenting, interviewing (self-administered), peer recruitment training, coupon issuance, compensation, and recruitment tracking. All functions were automated; data managers monitored recruitment, data collection, and payment and could be contacted by recruits as needed. Eligible participants were male, older than 15 years, resided in Thailand, and had anal sex with a man in the past 6 months. Recruits who resided in Bangkok were additionally invited to physically attend a participating health clinic of their choice for an HIV-related blood draw. Data were weighted to account for the complex sampling design.

**Results:**

The survey was implemented from February to June 2022; seeds (21 at start, 14 added later) were identified mostly through targeted web-based banner ads; coupon uptake was 45.1%. Of 2578 candidate recruits screened for eligibility, 2151 (83.4%) were eligible and 2142 (83.1%) enrolled. Almost all (2067/2578, 80.2%) completed the questionnaire; however, 318 survey records were removed from analysis as fraudulent enrollments. The final sample size was 1749, the maximum number of waves achieved was 191, and sampling covered all 6 geographic regions and 75 of 77 (97.4%) provinces; convergence was reached for several salient variables. The mean age was 20.5 (SD 4.0) years, and most (69.8%) had never tested for HIV before, with fear of stigma as the biggest reason (97.1%) for not having tested. Most (76.9%) had visited gay-focused physical venues several times a week. A condom was used in 97.6% of the last sex acts, 11.0% had purchased sex from other men (past 12 mo), 4.5% had sold sex to men (past 12 mo), and 95.3% had 3+ male sex partners (last 3 mo). No participant in Bangkok presented for a blood draw.

**Conclusions:**

We successfully conducted a web-based RDS survey among MSM in Thailand, covering nearly the entire country, although, as in physical RDS surveys, sampling was dominated by younger MSM. The survey also failed to collect biomarkers in Bangkok. Public health interventions should aim at increasing testing and addressing (the perception of) stigma.

## Introduction

The global share of new HIV infections due to key populations and their partners is steadily rising and was estimated at 70% in 2021 [1]. Key populations, including men who have sex with men (MSM), transgender persons, sex workers, people who inject drugs, and people in prisons and other enclosed settings, face extraordinarily high HIV acquisition risks [1], warranting intense HIV control efforts tailored to these populations. To inform programming and policy making, accurate HIV estimates at the population level are needed, in addition to monitoring and evaluation of programs. However, the lack of sampling frames for key populations poses a substantial challenge for surveillance and estimation, and necessitates work-arounds to generate population-level estimates, such as HIV prevalence, uptake of services, and progress toward the Joint United Nations Programme on HIV and AIDS (UNAIDS) 95-95-95 targets. Currently, the most frequently used sampling and analytic design to facilitate such population-level estimates is respondent-driven sampling (RDS) [2,3]. RDS represents an advanced version of snowball sampling, with refinements in the way data are collected and analyzed. Relying on the recruitment among socially connected peers, RDS surveys are typically conducted in a single location and rely on staffed survey offices for data collection.

In 2021, Thailand had 48.59 million internet users, representing 69.5% of the population, an increase of 7.4% from 2020. Additionally, there were 55.00 million social media users, accounting for 78.7% of the total population [4]. According to a survey conducted among gay men in Thailand (n=277), 78.7% of respondents reported using social network apps such as Facebook and Jack’d to find partners [5]. The rise of the internet and the increasing access to and time spent on the internet also open new possibilities for HIV surveillance, such as population size estimation [6,7] and surveys [8]. Similar to most health-related web-based surveys, web-based data collection among key populations primarily relies on samples based on self-selection and or unknown sampling probabilities [9,10]. Successfully implemented web-based RDS surveys among various populations [11,12], including MSM [13], remain sparse perhaps because building the required IT infrastructure can appear daunting. Still, web-based surveys facilitating population estimates are attractive for 2 principal reasons: the promise of large-scale surveys covering geographies not normally included in biobehavioral surveys [14] due to budgetary or sampling (rural areas) reasons and lower costs compared with a “brick-and-mortar” survey office. However, new challenges arise with web-based data collection such as fraudsters or duplicate enrollments due to the compensation paid out to recruits, as well as the impracticality of biomarker collection. For HIV-focused population surveys, biomarkers are often considered essential metrics facilitating estimates such as HIV prevalence, HIV status awareness, or viral load suppression.

Thailand faces a key population-dominated HIV epidemic with an estimated adult HIV prevalence of 1.0% [15]. Most new infections (52%) appear to arise from male-male sex [16]. Thailand has a long history of HIV surveillance, including time location sampling surveys for MSM and benefits from a robust case surveillance system [17]. A consensus meeting reviewing data from 2014 to 2018 concluded that HIV prevalence and incidence among MSM in Bangkok declined [18] but nevertheless remained high, with UNAIDS estimating HIV prevalence among MSM in Thailand at 11.9% [19].

This study aimed to evaluate and demonstrate the feasibility of a web-based RDS survey that could be implemented as a routine surveillance system in the country. The goal was to enhance surveillance efforts to reach hard-to-access populations in Thailand, where there is a high rate of social media use. If this study proved that web-based surveys are feasible in Thailand, it could establish a standardized web-based surveillance model for the country. This would provide valuable data for effectively addressing the HIV/AIDS epidemic.

We piloted a web-based RDS survey with biomarker collection among MSM in Thailand. In this paper, we report on the survey’s design, implementation, and findings.

## Methods

### Survey Setting and Design

We conducted a web-based RDS survey among MSM in all of Thailand, dubbed the Kai Noi survey. The survey was implemented by staff from the Division of Epidemiology (Ministry of Public Health, Thailand) with funding support and technical assistance from the US Centers for Disease Control and Prevention (CDC). The RDS survey system’s digital architecture is explained in detail in “A Web-Based, Respondent-Driven Sampling Survey Among Men Who Have Sex With Men (Kai Noi): Description of Methods and Characteristics” [20]. In short, a website was constructed to fully function like a conventional RDS survey office, including coupon verification, eligibility screening, consenting, interviewing (self-administered), peer recruitment training, coupon issuance, compensation, and recruitment tracking. All these functions were automated; even so, a data manager monitored recruitment and data collection, answered anecdotal questions from recruits (via the web or by phone), and supervised the execution of payments. Additional survey monitoring included coupon uptake and sampling speed, as well as convergence and bottleneck graphs.

Eligible participants and seeds (ie, participants who started the peer referral-based sampling process) were born male, identified as male, were 15 years or older, had anal sex with another man in the last 6 months, had not previously participated in this survey, resided in Thailand, could read Thai, and presented a valid electronic coupon (eCoupon; except seeds). Seeds were solicited through banner ads or micromessaging in gay-friendly social media (pages) and were sought from each of Thailand’s 6 geographic regions to ensure geographic diversity and social distance. Eligible and consenting seeds started the recruitment process using eCoupons that could be distributed through various social media, such as chat applications, or SMS. eCoupons were labeled with a nonserial coupon ID and displayed a web address linking to the survey website [21].

### Interview Data Collection

The self-administered questionnaire was short and covered basic demographics, personal network size (used to inform sampling weights), recruiter-recruit relationship, cyberspace use, HIV service uptake, and HIV-related risk behaviors. Preprogrammed data checks and skip patterns were embedded. An attention filter question was inserted in about the middle of the questionnaire (ie, a question informing the recruit that this question probes whether he pays attention and asking him to select a specific response value). Time points at which sentinel events occurred were recorded for documentation and monitoring.

### Data Analysis

RDS Analyst [22] software was used to derive weighted estimates, adjusting for the complex sampling design using Gile’s SS estimator and a national MSM size estimate of 588,000 (AIDS Epidemic Model, Thailand data sheet, unpublished data, 2022). We conducted an analysis to determine the independence between recruiters and recruits by examining the tendency for people to form social ties with others who share similar traits, which is known as homophily. The analysis considered two scenarios: (1) when the outcome clusters by network or (2) when network members cluster in space and the outcome is spatially clustered. We also performed a convergence analysis to assess whether the sample size in this study was sufficient to achieve results in the desired characteristics, and to determine if increasing the sample size further would lead to no changes in the results. Additionally, we conducted a univariate analysis, stratified by age or other characteristics as needed.

### Biomarkers

Following interview completion, participants in Bangkok only were also offered to attend 1 of 10 routine health clinics of their choice for a blood draw. A venous blood sample would be tested for HIV serology (using the prevailing HIV rapid test algorithm), HIV recency, hepatitis B and C, and syphilis. At the clinic, recruits were to present their phone number and undergo in-person consenting at the clinic.

### Ethical Considerations

The survey protocol was approved by the Ethical Review Committee of the MOPH, and was approved by the CDC in Atlanta, United States. Informed consent was obtained electronically, separately for the interview and biomarker testing. A copy of the consent could be downloaded by the participant. Personal identifiers, including cell phone numbers and IP addresses, were collected and converted to unique anonymous codes. All records were labeled using the eCoupon ID number. Participants could call survey staff with questions. At the clinic, recruits were to be pre- and posttest counseled and, as warranted, initiated on HIV preexposure prophylaxis (PrEP) or treatment, as well as provided treatment or care for syphilis and viral hepatitis as per MOPH guidelines. Recruits were compensated for survey enrollment and interviews (US $8.80); peer recruitment (US $4.40) for each successfully recruited peer, up to a maximum of US $8.80; and biomarker collection at a clinic (US $14.70).

The research project was approved by the Ethics Committee for Research in Human Subjects, Department of Disease Control, Thailand (FWA number 00013622). The US CDC determined that it was research with CDC investigators not engaged (Project ID 0900f3eb81afe111).

### Survey Costs

The costs for preparing and implementing the web-based RDS survey included meetings and consultations to design the survey and develop the protocol, creation of the Kai Noi website, graphic design, hardware equipment, training, testing, server rental fees, volunteer compensation, web-based advertising, and compensation paid to survey participants, resulting in total costs of US $36,046 or US $22 per participant (n=1643).

## Results

### Sampling

The Kai Noi survey was implemented from February to June 2022. A total of 673 self-selected web-based candidate seeds volunteered to initiate the eligibility screening; of these, 390 (57.9%) were deemed ineligible and a further 248 (36.8%) did not complete the eligibility interview. The remaining 35 (5.2%) candidates were eligible and all consented to enroll. We initiated peer recruitment with 21 of the 35 candidate seeds and added the remaining 14 seeds during the sampling period. Of the 35 seeds, 13 (31.1%) successfully recruited at least 1 peer, and the remaining 22 did not successfully recruit any eligible peers.

Each seed and recruit was issued 3 eCoupons for peer recruitment. Of 6207 eCoupons issued, 3000 (45.1%) were redeemed. The mean time between eCoupon issuance and redemption was 9.7 (SD 32.7) hours. A further 1008 eCoupons were presented that had already been redeemed and were therefore deemed invalid; in addition, 243 eCoupon IDs logged in by candidate participants were deemed invalid as they never been had issued. Almost half (48%) of candidate recruits logged into the survey website between 5 PM and 11:59 PM (GMT +7).

A total of 2578 candidate recruits (including seeds) with valid eCoupons and unique phone numbers were admitted to screening for eligibility, of which 136 (5.3%) were deemed ineligible due to reasons related to sex or gender (36.1%), age (5.9%) or lack of recent same-sex behavior (58.0%); a further 291 (11.3%) did not complete the eligibility screening questionnaire. The remaining 2151 (83.4%) were deemed eligible. Of these, 2142 (83.1%) agreed to participate, and of these, 2067 (80.2%) completed the questionnaire. During the sampling period, survey staff noticed potentially fraudulent behavior, confirmed it, and subsequently identified 318 survey records as repeat enrollments and hence fraudulent. These records, along with their redeemed and unredeemed eCoupons were removed from the analysis, leaving 1749 valid survey records. The mean social network size (based on having been in contact with known and likely eligible peers within the last 7 days) among enrolled survey participants was 2.3 (IQR 1-3). We excluded 103 records stemming from participants who did not pass the main interview’s attention filter question, leaving a final sample size of 1643 for the main interview data analysis. This sample restriction did not apply to the eligibility screening data (sex, age, residence, and same-sex sexual behavior) which was administered separately and prior to the main interview.

Table 1 displays seed-related sampling characteristics. For the sample of 1749 recruits, the maximum number of waves achieved was 191; with 1 seed accounting for 1549 (88.6%) recruits sampled. Recruits were sampled from all 6 regions (Table 2) and from 75 of 77 (97.4%) provinces (data not shown).

**Figure 1.**
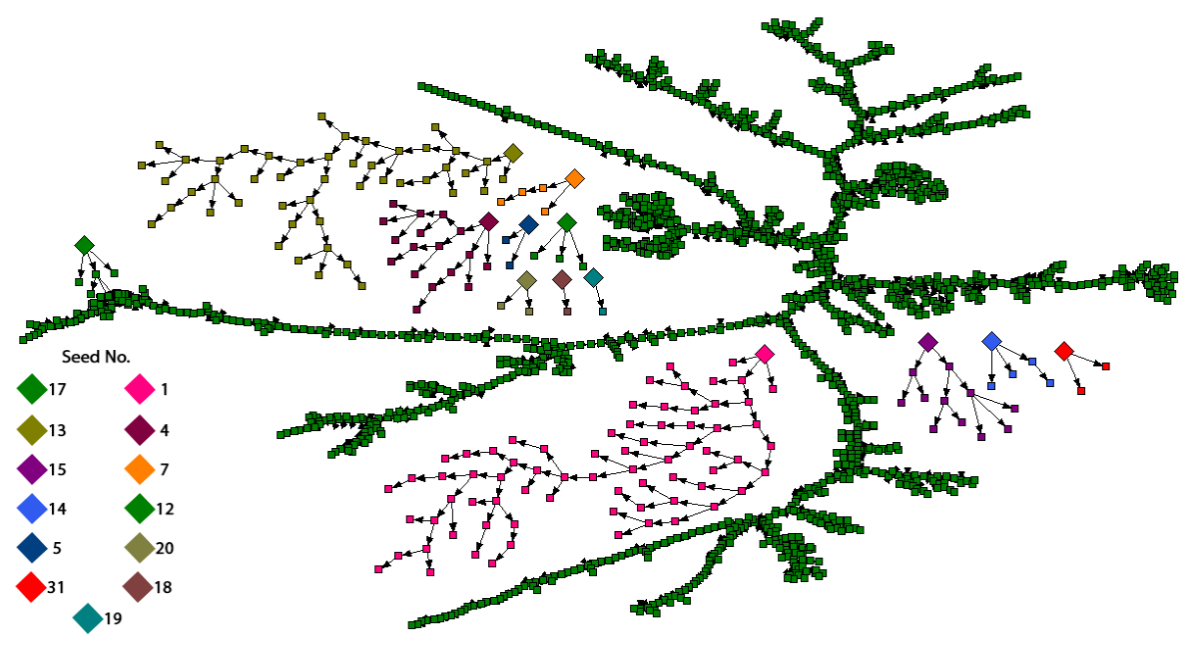
Recruitment tree by seed.

**Figure 2.**
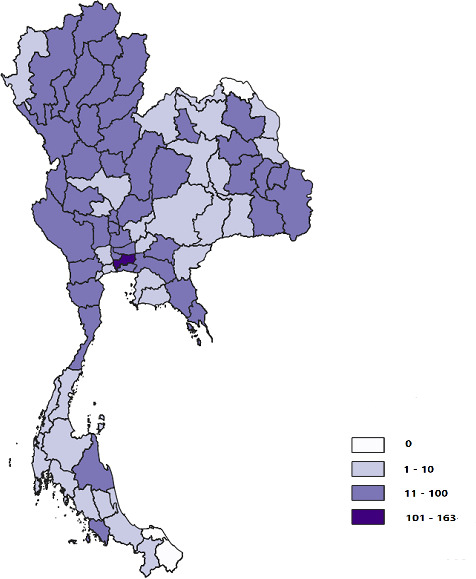
Distribution of enrollments by residence (N=1749) across 77 provinces in Thailand, displayed on a map with color-scale gradient based on the number of participants in each province.

Figure 1 displays the distribution of recruitment by seed (n=1749); Figure 2 shows the distribution of survey enrollments across Thailand.

**Table 1.** Description of recruitment by seed.

Characteristic		Age (years)	Degree	Province (seed)	Max number of waves	Number of recruits (including seeds)	Percentage of sample (%)
Seed number							
	1	31	10	Bangkok	18	73	4.2
	4	20	8	Payao	4	18	1.0
	5	36	20	Bangkok	1	3	0.2
	7	22	2	Ratchaburi	3	5	0.3
	12	25	5	Bangkok	1	4	0.2
	13	30	5	Nongbualamphu	15	48	2.7
	14	39	10	Chiang Mai	2	5	0.3
	15	31	5	Chiang Rai	3	12	0.7
	17	19	5	Khon Khaen	191	1549	88.6
	18	31	6	Ang thong	1	2	0.1
	19	47	2	Phetchaburi	1	2	0.1
	28	28	3	Ratchaburi	1	3	0.2
	31	30	14	Nonthaburi	1	3	0.2
Total (excluding nonproductive seeds)		—^a^	—	—	—	1727	98.7
Number of nonproductive seeds		—	—	—	—	22	1.3
Grand total		—	—	—	—	1749	100

a Not applicable.

**Table 2.** Demographic characteristics of men who have sex with men sampling. Sample size for age data is 1749, as age data were collected in the eligibility interview, separate from the main interview, with the embedded attention filter question that led to the exclusion of 103 records from the main interview, leading to a final sample size of 1643 for data analysis.

Characteristics	Unweighted, n (%)		Weighted (%)		
			Point	L95% CI^a^	U95% CI^b^
Sampling by region (n=1749)					
North	336 (19.2)		20.1	17.4	22.9
Central	653 (37.3)		37.1	33.5	40.8
Northeast	378 (21.6)		22.2	19.3	25.0
West	220 (12.6)		12.7	10.4	15.0
East	74 (4.2)		4.1	3.0	5.1
South	88 (5.0)		3.8	2.4	5.2
Age distribution (years), (n=1749)					
15‐19	807 (46.1)		59.3	51.2	67.4
20‐24	552 (31.6)		25.9	21.4	30.4
25‐29	315 (18.0)		11.5	8.0	15.0
30+	75 (14.3)		3.3	1.45	5.3
Highest education (n=1643)					
No schooling	14 (0.9)		0.5	0.0	1.7
Secondary school	39 (2.4)		2.7	0.8	4.5
High school	1138 (69.3)		83.0	76.4	89.6
Diploma	98 (6.0)		3.8	2.3	5.2
Bachelor	233 (14.2)		6.1	2.9	9.3
Master and higher	121 (7.4)		3.9	1.3	6.6
Work (n=1643)					
Student	927 (56.4)		70.6	58.3	82.8
Employee	320 (19.5)		16.9	9.1	24.6
Own business	99 (6.0)		2.7	1.3	4.1
Laborer	97 (5.9)		3.7	1.4	6.0
Office worker	67 (4.1)		2.0	0.9	3.2
Unemployed	60 (3.7)		1.3	0.0	3.4
Merchant	35 (2.1)		1.0	0.4	1.7
Government employee	18 (1.1)		0.7	0.2	1.1
Other	20 (1.2)		0.9	0.4	1.4
Reasons to use the internet (multiple choice format)					
To find sex	1451 (88.3)		89.3	85.9	92.6
To sell or buy sex	87 (5.6)		1.9	1.4	2.4
To learn about HIV or use HIV services	35 (2.1)		0.6	0.3	0.9
To pay or receive money	259 (15.8)		16.1	12.0	20.1
To play games	1006 (61.2)		68.1	61.3	74.9
Social app use (multiple choice format)					
BlueD	1103 (53.2)		77.6	64.6	90.5
Grindr	75 (3.6)		3.0	1.2	4.8
Hornet	92 (4.4)		2.6	1.4	3.9
Jack’d	71 (3.4)		2.1	0.6	3.5
Gay Romeo	286 (13.8)		9.0	3.6	14.4
Tinder	445 (21.5)		16.7	11.6	21.8
Facebook	779 (47.4)		39.5	32.3	46.6
Line	555 (33.8)		20.8	14.6	27.0
Instagram	417 (25.4)		17.0	13.0	21.1
Twitter	494 (30.1)		18.5	14.7	22.2

a L95%CI: lower 95% CI.

b U95%CI: upper 95% CI.

### Homophily and Convergence

#### Homophily

Table 3 displays homophily by select characteristics. We observed homophily for all 4 traits examined, with substantial homophily for age, region of residence, and timing of last HIV test. Recruiters were almost twice as likely to recruit peers sharing their age (group), region of residence, or HIV status as expected for random recruitment. There was little homophily for condom use.

**Table 3.** Homophily by select characteristics.

Trait	Categories	Homophily	*P* value
Age (years)	15‐19, 20‐24, 25‐34, and 35+	1.97	<.001
Residence	Regions 1 through 6	1.86	<.001
Timing of last HIV test	Last 12 months, >12 months ago, never	1.98	<.001
Condom use at last sex	Yes, no	1.08	<.001

#### Convergence

**Figure 3.**
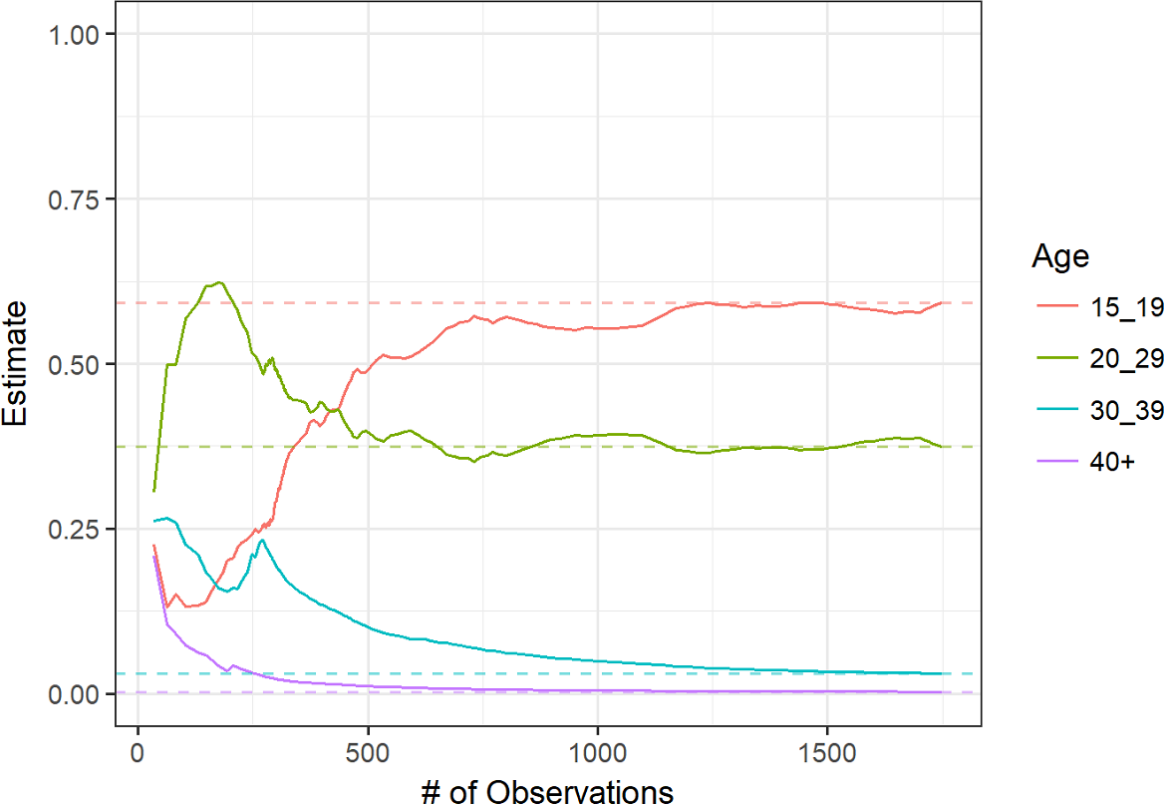
Convergence graph for age.

**Figure 4.**
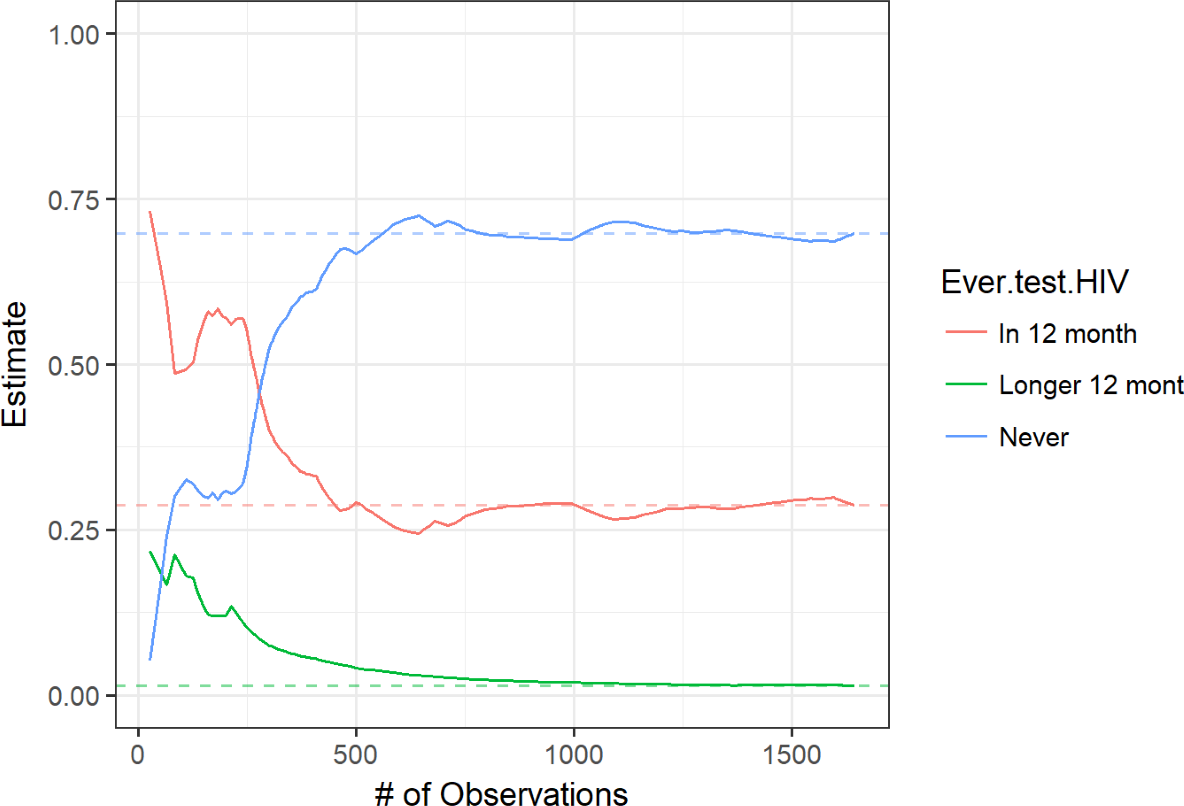
Convergence graph for HIV testing.

**Figure 5.**
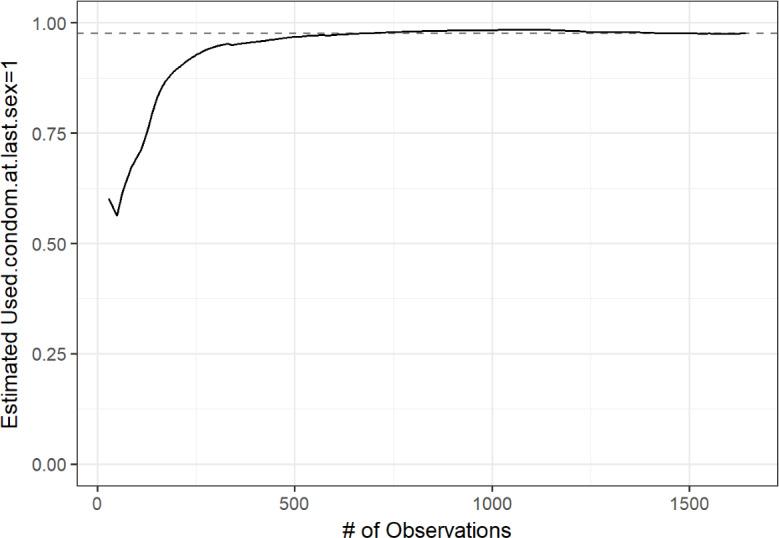
Convergence graph for condom use at last sex.

**Figure 6.**
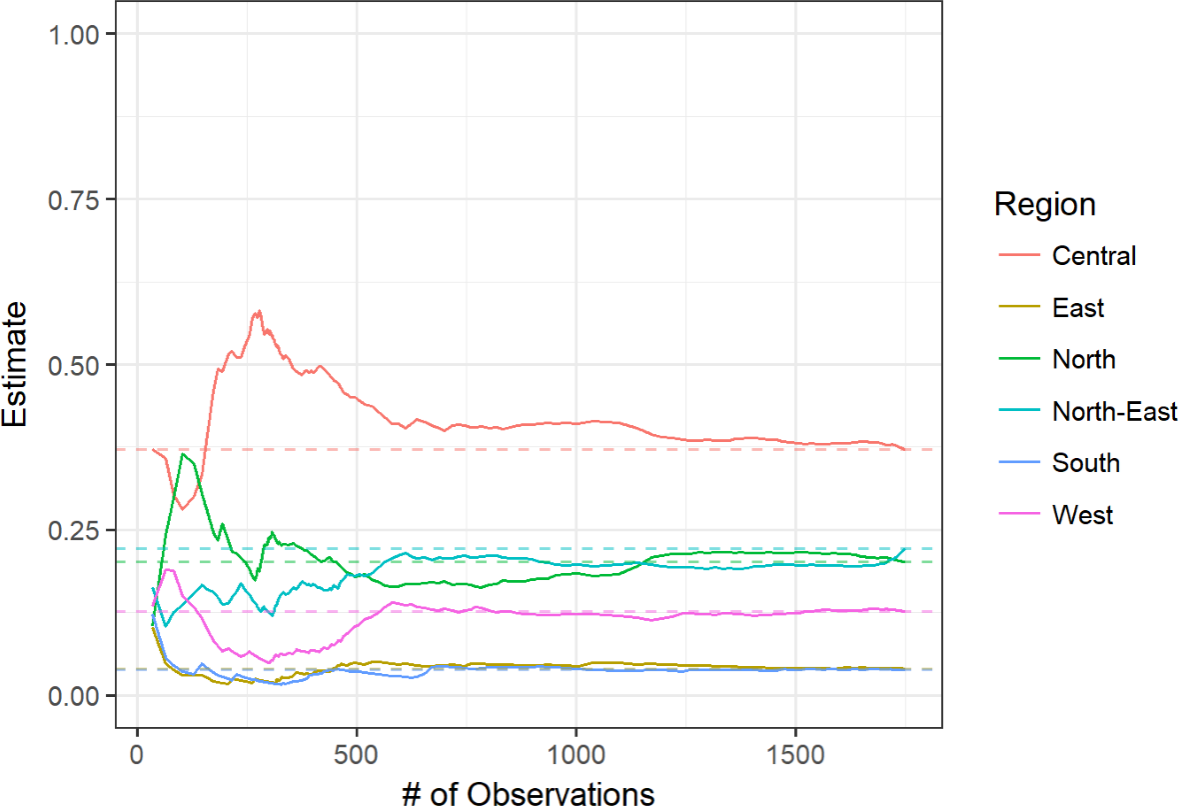
Convergence graph for region.

Figures 3-6 display convergence graphs for select characteristics. Convergence for age and region was reached after approximately 1000 participants, and that for (regional) residence and condom use was seemingly reached earlier. The interpretability of bottleneck graphs was greatly diminished as 1 seed’s recruitment tree accounted for 89% of the total sample, hence no bottleneck graphs are displayed here.

### MSM Characteristics

#### Demographics

Table 2 displays the demographic characteristics of MSM sampling covering all 6 regions. The mean age was 20.5 (SD 4.0, median 20, IQR 19‐24, range 15‐56) years. Most had a high school education or higher and were currently in school. The majority (70.6%) used the internet to find sex or play games; BlueD was the most used social media app (77.6%).

#### HIV-Related Behaviors and Service Uptake

Most (76.9%) MSM visited gay-focused venues several times a week, 11.0% had purchased sex from men in the past 12 months, and 4.5% had sold sex to men in the past 12 months (Table 4). Almost all (95.3%) had more than 2 sex partners in the last 3 months, and almost all (97.6%) used a condom at their last sex act. Few (4.0%) had sex with a woman in the last 12 months or had injected drugs in the last 12 months (0.3%). Two-thirds (68.9%) had never tested for HIV, almost always out of fear of being stigmatized (97.1%). Current PrEP use was high (89.4%).

**Table 4.** Risk behaviors and service uptake. Only 1 participant stated that his last test result was HIV-positive (data not shown).

Risk behavior (n=1643)	Unweighted, n (%)		Weighted (%)		
			Point	L95% CI^a^	U95% CI^b^
Visits to gay-friendly venues (eg, saunas, clubs, bath houses) several times a week	1095 (66.6)		76.9	70.5	83.3
Bought sex from other men (last 12 mo)	376 (22.9)		11.0	7.2	14.8
Sold sex to other men (last 12 mo)	211 (12.8)		4.5	2.7	6.2
Two or more male sex partners (last 3 mo)	1555 (95.6)		95.3	93.1	97.5
Condom use at last same sex act	1554 (94.6)		97.6	95.8	99.4
Sex with women (last 12 mo)	108 (6.6)		4.0	1.8	6.2
Injecting drug use (last 12 mo)	8 (0.5)		0.3	0.0	0.6
Service uptake					
Timing of last HIV test					
In the last 12 months	675 (41.1)		28.7	11.7	45.7
Longer than 12 months ago	54 (3.3)		1.5	0.4	2.6
Never tested for HIV	914 (55.6)		69.8	52.1	87.5
Reason for never having tested for HIV (n=914)					
Don’t feel at risk	40 (4.4)		1.6	0.0	5.7
Fear of positive result	14 (1.5)		0.7	0.1	1.3
No money to get tested	12 (1.3)		0.1	0.0	0.3
No time to get tested	16 (1.8)		0.4	0.0	1.4
Fear of stigma	832 (91.0)		97.1	91.9	100.0
Preexposure prophylaxis (PrEP; restricted to those who reported previous HIV negative test result, n=728)					
Have heard about PrEP	676 (92.9)		95.6	88.2	100.0
Currently using PrEP	612 (90.5)		89.4	80.1	98.8
Among those who are not currently using PrEP (n=64)					
Used it in last 6 months	4 (6.3)		2.6	1.2	3.9
Used it >6 months ago	7 (10.9)		7.5	2.6	12.5
Never used it	53 (82.8)		89.9	84.7	95.1
Among those never used PrEP (main reason; n=53)					
Don’t know about it	1 (1.9)		0.3	0.0	0.7
Don’t know where to get prep	4 (7.5)		5.4	1.7	9.1
Embarrassed to ask for it	3 (5.7)		1.6	0.0	4.3
Don’t feel at risk	36 (67.9)		81.2	64.9	97.6
Afraid of side effects	4 (7.5)		4.9	0.0	11.5
Don’t want others to know	5 (9.4)		6.5	0.0	16.9

a L95% CI: lower 95% CI.

b U95% CI: upper 95% CI.

### Biomarkers

There were 144 participants who resided in Bangkok. All of them were invited to attend 1 of 10 clinics to provide a blood sample; 82% agreed online to do so. However, no recruit presented at any of the 10 clinics during the survey, and no biomarker data were collected.

## Discussion

We successfully conducted a web-based RDS survey among MSM in Thailand, adding to the still small body of literature on web-based probability sampling among key populations. The survey covered nearly the entire country, with no region or province dominating recruitment, and reached convergence for salient variables. However, our goal of collecting biomarkers was not achieved, and the fraudulent enrollments are a reminder that financial compensation for web-based data collection may quickly attract fake (duplicate) enrollments.

As seen with other conventional RDS surveys, sampling was dominated by younger, relatively well-educated MSM. A large proportion of MSM uses the internet to “find” (mostly noncommercial) sex, which suggests that the web may be a useful tool to engage MSM for safe sex and HIV prevention messages. Of potential importance are the estimates for social media apps as these estimates can inform the reach of public health messaging in cyberspace across various apps. The most frequently used app appears to be BlueD, followed by Facebook. The estimated HIV testing uptake was low with less than a third ever having tested for HIV, even considering the age distribution skewed toward younger ages. Still, restricting our analysis to just the 4 provinces overlapping with the 2020 MSM biobehavioral surveys (Bangkok, Chiang Mai, Chonburi, and Phuket) [23], the lifetime HIV testing uptake estimates appear similar (web-based RDS: 63.8%; biobehavioral surveys: 52.9%). “Fear of stigma” was by far the most frequent reason given for not having tested, an important finding for Thailand’s HIV testing services. The low rate of HIV testing is contrasted by high proportions of MSM reporting condom use (at last sex) and PrEP use.

This survey also confirms that many (young) MSM continue to use offline venues such as saunas, bars, and clubs several times a week. HIV risk behaviors were confirmed in several dimensions, including buying and selling sex, as well as having multiple sex partners (for most MSM, 3 to 5 partners in the preceding 3 months). A positive finding was the very high proportion of condomized sex acts (98%).

Several substantial challenges and limitations must be noted, including a very low proportion (n=1) of participants reporting an HIV-positive status, which is unlikely for a sample size of 1643 and suggests that some participants were reluctant to report an HIV-positive status. Convergence for the traits examined was only reached after hundreds of recruits had been sampled, perhaps due to the very large sampling area. Because a single seed accounted for a very large proportion of our sample, we could not assess bottlenecks; at the same time, this observation supports the assumption that Thai MSM form a single network. The self-reported degrees were somewhat lower than expected, raising the possibility of inaccuracies. Further, our survey was exposed to fraud, an unsurprising risk given that compensation in RDS surveys is common. Survey staff suspected and eventually confirmed large-scale fraud when a participant contacted the survey staff with questions related to compensation that appeared odd to our staff. Over 300 fraudulent enrollments were detected despite multiple security measures to prevent such fraud. From an analysis point of view, the fraud cases in our survey represented mostly a loss of funds (paid-out compensation) and, assuming we identified all fraudulent “participants,” did not do lasting damage to the dataset, as we simply removed fraudulent enrollments from the dataset for analysis. Further, because (unsurprisingly) none of the fraudulent “recruits” peer-referred valid recruits, our dataset did not experience breaks in the recruitment chains. Web-based RDS surveys are well advised to maximize antifraud measures and to monitor proactively to detect fraudulent enrollments. Our national-level survey may mask important differences across localities and hence may not make local surveys redundant. Our biggest limitation is perhaps the failure to collect blood samples from recruits residing in greater Bangkok. The reasons for this are unclear, as most participants residing in Bangkok indicated via the web that they would be willing to present at a clinic, in exchange for additional financial compensation. Alternative designs involving compensation (all-in-one compensation paid only after the blood draw), blood collection (mailing kits for self-collection), or testing (mailing self-tests) should be evaluated. Of note, our survey was launched during the peak period of COVID-19 transmission in Thailand. The number of persons testing for HIV at the same 10 Bangkok sites as in our survey decreased from 82,054 in 2020 to 60,124 in 2022 during the fifth wave of Omicron COVID-19 [24].

The strengths of our survey included a fully automated RDS system that fulfilled all essential functions of a “brick-and-mortar” RDS survey office, including checking coupon validity, eligibility screening, consent, interview, coupon issuance, peer recruitment training, and compensation, promising and delivering efficiency and resource savings. The web-based design also facilitated a sampling process that covered the entire country. The code for our web-based RDS system is available from the investigators and can be redeployed with minimal preparatory effort within Thailand, that is, it can easily be adapted for other target populations. The code could also be used outside Thailand, assuming IT expertise is available to adapt the survey to a different geographic setting.

While the up-front coding work to construct a virtual RDS office can be substantial, other tasks in survey preparation become obsolete, such as securing physical survey office space, or may pose substantially smaller burdens, such as the number of staff or computer and other hardware equipment needed.

The need for key population–specific population-level estimates will keep increasing as countries monitor their progress toward equitable HIV epidemic control. With internet use becoming more ubiquitous, there is potential for web-based RDS surveys to help fill these data needs. More operational research is needed though in refining web-based RDS methods, including fraud prevention and detection, facilitating a more age-diverse sample, and bridging the online-offline gap in collecting biomarker data.

## Abbreviations

CDC: Centers for Disease Control and Prevention
eCoupon: electronic coupon
MSM: men who have sex with men
PrEP: preexposure prophylaxis
RDS: respondent-driven sampling
UNAIDS: Joint United Nations Programme on HIV and AIDS
